# 

**Published:** 1924-04

**Authors:** 


					APRIL, 1924.
v?l- XLI.
No. 152.
THE
BRISTOL
medico-chirurgical
1. V A
runr.Tsriun undicii tub auspigiis or// - '? Lrn,
if "<?/?
THE BRISTOL MEDICO-CHIRURGlfcAL SOCIETY.
Editor: P. WATSON-WILLIAM
WITH WHOM ABE ASSOCIATED
JAMES SWAIN, C.B., C.B.E., M.S., M.I).,
J. LACY FIRTH, M.S., M.D., CAREY COOMBS, M.D.
J. A. NIXON, C.M.G., M.D., Assistant Editor.
Editorial Secretary: A. L. FLEMMING, M.B., B.Ch.
^ Bristol: j. w. arrowsmith ltd., h quay street.
xdox ; j w Arrowsmith (London) Ltd., 6 Upper Bedford Place,
Russell Square.
Price Three Shillings net.

				

